# Hypertension associated with venous thromboembolism in patients with newly diagnosed lung cancer

**DOI:** 10.1038/srep19603

**Published:** 2016-01-22

**Authors:** Yuhui Zhang, Yuanhua Yang, Wenhui Chen, Lirong Liang, Zhenguo Zhai, Lijuan Guo, Chen Wang, China Venous Thromboembolism (VTE) Study Group, Li Zhang, Qixia Xu, Luning Jiang, Xinhong Zhang

**Affiliations:** 1 Beijing Institute of Respiratory Medicine, Beijing Key Laboratory of Respiratory and Pulmonary Circulation Disorders, Beijing Chao-Yang Hospital, Capital Medical University, Beijing 100020, China; 2 National Clinical Research Center of Respiratory Medicine, China-Japan Friendship Hospital, Beijing 100029, China; 3 Department of Respiratory Medicine, Peking Union Medical College Hospital, Chinese Academy of Medical Sciences, Beijing, 100730, China; 4 Department of Respiratory and Critical Care Medicine, the First Affiliated Hospital of Bengbu Medical College, Bengbu, 233004, China; 5 Department of Respiratory Medicine, The Affiliated Hospital of Jining Medical College, Jining, 272001, China; 6 Department of Oncology, The Navy General Hospital of PLA, Beijing, 100048, China

## Abstract

The aim of this study was to evaluate associations between cardiovascular disease (CVD) risk factors and the occurrence of venous thromboembolism (VTE) in patients with lung cancer that might help estimate an individual’s risk for VTE. A total of 632 unselected patients with newly diagnosed lung cancer were investigated for VTE within the three months prior to recruitment, and their major CVD risk factors were assessed at the baseline examination. Eighty-six of the 632 (13.6%) developed a VTE event. Multivariate logistic regression analysis, including age, sex, smoking, body mass index, diabetes, dyslipidemia, hypertension and white blood cell count, found that hypertension (OR 1.8; 95% CI 1.0–3.3) and leukocytosis (OR 2.7; 95% CI 1.5–4.8) were significantly associated with VTE in different tumor histology models and that hypertension (OR 1.9; 95% CI 1.1–3.4) and leukocytosis (OR 2.7; 95% CI 1.5–4.7) were also significantly associated with VTE in different tumor stage models. Leukocytosis was linearly associated with hypertension and VTE (*P* for trend = 0.006), and the ORs for VTE increased with leukocytosis (all *P* for trend <0.05). In conclusion, hypertension increased the risk of VTE in patients with newly diagnosed lung cancer, which may be mediated by the presence of inflammation.

Venous thromboembolism (VTE) is one of the highest complications in patients with solid tumor ,especially in lung cancer^1^,^2^,^3^. Classic risk factors for cancer-associated VTE include aging, poor performance status, prior history of VTE, histology of cancer, origin of cancer, chemotherapy, surgery, and other treatment-related factors^1^,^4^,^5^,^6^,^7^. Although the risk factors potentially responsible for this disorder are identifiable in most patients, cancer-associated VTE remains unexplained in some patients.

Cardiovascular disease (CVD) risk factors, such as smoking, obesity, hypertension, dyslipidemia, and diabetes, often coexist with cancer. The risk of arterial thrombosis in patients with major CVD risk factors is most likely mediated by the presence of an inflammatory state and hypercoagulability^8^. Both increased inflammation and coagulation may predispose these patients to develop VTE events^9^. Furthermore, metabolic syndrome increases the risk of developing multiple types of cancer and VTE^10^,^11^,^12^,^13^. We hypothesized that cancer patients with CVD risk factors may be at increased risk of VTE. To test this hypothesis, we evaluated associations between major CVD risk factors and occurrence of VTE in patients with newly diagnosed lung cancer.

## Results

### Patient characteristics

**Figure 1:**
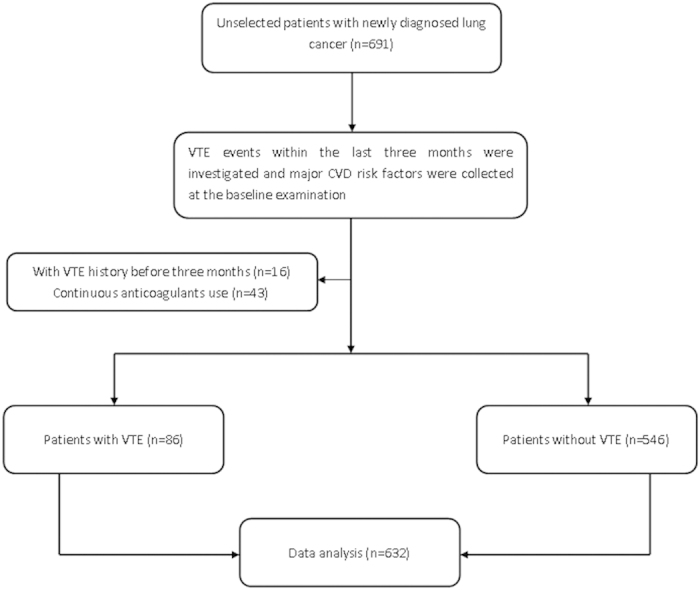
Study flow diagram. Abbreviations: VTE = venous thromboembolism; CVD = cardiovascular disease.

A total of 691 unselected patients with newly diagnosed lung cancer were enrolled in this study. Sixteen patients were excluded because they had a history of deep vein thrombosis (DVT) or pulmonary embolism (PE) more than three months before recruitment. Another forty-three patients were excluded because they were continuously taking anticoagulants. In the end, 632 eligible patients were included in our study (Fig. 1).

The 632 included lung cancer patients had a median age of 63.5 years, and 71.7% of the patients were male. The baseline demographic and clinical characteristics of the investigated study population are listed in Table 1. To analyze non-small cell lung cancer and small cell lung cancer collectively, the tumors were histologically categorized as adenocarcinoma or non-adenocarcinoma (all lung cancers with the exception of adenocarcinoma), and the tumor stage was categorized as localized stage (confined to ipsilateral hemithorax) or distant metastasis. The study population consisted of patients with adenocarcinoma (n = 295) and patients with non-adenocarcinoma (n = 337). Distant metastases were found in 276 patients (43.7%).

### VTE events

Overall, 86 of the 632 patients (13.6%) experienced a VTE event. 40 patients (6.3%) developed lower-extremity DVT alone, 32 patients (5.1%) developed PE alone, and 14 patients (2.2%) developed both DVT and PE.

### CVD factors and risk of VTE

We evaluated the associations between major CVD risk factors (age, sex, smoking, body mass index, diabetes, hypertension, total cholesterol, triglycerides, and white blood cell count) and risk of VTE in our study. VTE occurred more frequently in patients with hypertension or leukocytosis than in patients without them (*P* < 0.05 for both). Detailed information for the patients with and without VTE is provided in Table 2.

Subsequently, we performed a multivariate logistic regression analysis (model 1) that included the age, sex, smoking, body mass index, diabetes, hypertension, dyslipidemia, and white blood cell count of newly diagnosed lung cancer patients with different tumor histologies (adenocarcinoma vs. non-adenocarcinoma) to identify factors associated with VTE (Table 3). Hypertension (vs. without, odds ratio [OR] 1.8; 95% CI 1.0–3.3; P = 0.041) and leukocytosis (vs. WBC < 10 × 10^9^/L, OR 2.7; 95% CI 1.5–4.8; *P* = 0.001) were significantly associated with VTE. Furthermore, we performed a multivariate logistic regression analysis (model 2) that included the age, sex, smoking, body mass index, diabetes, hypertension, dyslipidemia, and white blood cell count of newly diagnosed lung cancer patients with different tumor stages (localized stage vs. distant metastasis) to identify factors associated with VTE (Table 4). Hypertension (vs. without, OR 1.9; 95% CI 1.1–3.4; *P* = 0.029) and leukocytosis (vs. WBC < 10 × 10^9^/L, OR 2.7; 95% CI 1.5–4.7; *P* = 0.001) were also significantly associated with VTE.

Finally, we analyzed the associations of leukocytosis and hypertension with VTE. The patients were classified as four subgroups (no hypertension and no VTE, only hypertension, only VTE, or both hypertension and VTE) according to the inflammation load. The rate of leukocytosis was significantly different in the four subgroups (*P* = 0.04) and linearly associated with hypertension and VTE (*P* for trend = 0.006). The ORs for VTE risk increased among the four subgroups (all *P* for trend < 0.05; Table 5).

## Discussion

In the study population of newly diagnosed lung cancer patients, hypertension was associated with risk of VTE, which may be mediated through inflammation. Age, sex, smoking, body mass index, diabetes, and dyslipidemia were not associated with VTE risk. The same results were found for patients with different tumor histologies and tumor stages.

Whether CVD risk factors increase VTE risk has been a focus of many cohort and case-control studies^14^,^15^,^16^,^17^. Obesity has been demonstrated to be an independent risk factor of a VTE event^18^,^19^,^20^,^21^, whereas conflicting results have been reported for smoking, hypertension, dyslipidemia, and diabetes^8^,^20^,^21^. However, we were not able to confirm an association between high body mass index and VTE. In our study, the 632 lung cancer patients included in this study had a lower mean body mass index of 23.3 (kg/m^2^) than the patients included in a previous study^19^, and 43.7% of the patients had distant metastases. The risk of obesity may be weakened due to weight loss and malnutrition in advanced lung cancer patients with tumor progression and metastasis.

Most studies agree that current smoking is associated with increased VTE risk^19^,^20^,^22^,^23^,^24^,^25^. Biologically, the association of smoking with VTE may be mediated through hypercoagulability and impaired fibrinolysis^22^,^26^. A Danish study found a dose-response relationship between current smoking and VTE risk. Former smokers have the same risk of VTE as patients who have never smoked, indicating the acute effects of smoking and underscoring the potential benefits of smoking cessation^23^. However, our study failed to show an association between smoking with VTE, which is in agreement with the results of some previous studies^18^,^27^. Smoking contributes to the development of cancer. Regarding smoking history in our study, 15.2% of the patients were former smokers, 41% of the patients were current smokers, and 43.8% of patients were non-smokers. The risk of smoking may be underestimated because of the pooling of ex-smokers with current smokers, a lack of distinction between light and heavy smokers, and a shorter follow-up time in this study.

The roles of diabetes and dyslipidemia in the pathogenesis of VTE are controversial^8^,^18^,^19^,^21^,^22^,^28^,^29^,^30^. Our analyses showed no correlation between diabetes or dyslipidemia and risk of VTE. The findings of our study are in agreement with published reports^8^,^19^,^21^,^22^,^29^. In this study, 7.8% and 3.3% of the patients experienced diabetes and dyslipidemia, respectively, and these rates are less than the rates obtained in previous studies. This difference is also likely because our study population consists of advanced lung cancer patients whose tumor growth and metastasis may deteriorate their nutritional status.

Interestingly, our findings corroborate the documented finding that hypertension is associated with increased VTE risk. The ORs for hypertension were less robust than those reported for established major risk factors for cancer-associated VTE, such as tumor histology and tumor stage. However, their coexistence increased the risk of VTE additionally. This result supported the previous finding that hypertension was associated with increased risk of VTE^15^,^22^. Hypertension was a common comorbidity in patients with lung cancer because 16.6% of the patients in our study had hypertension. The link between hypertension and cancer-associated VTE has received relatively little attention to date. Systemic low-grade inflammation, which was measured based on the level of leukocytes, is involved in the development of atherosclerosis and coronary heart disease, and leukocytosis may be a predictor of cardiovascular events^31^,^32^,^33^. We analyzed the association of leukocytosis and hypertension with VTE. Leukocytosis was linearly associated with hypertension and VTE (*P* for trend = 0.006). The ORs for VTE increased with leukocytosis (all *P* for trend < 0.05). Hypertension was likely associated with disorders in the system of blood hemostasis, endothelial dysfunction and vascular inflammation, which resulted in an increased risk of thrombosis.

Our study has some limitations. First, although this study was based on only population of lung cancer patients, it was a short-term survey. Second, the larger sample size is needed to provide sufficient power to detect associations of CVD risk factors and VTE risk. Finally, our study lies in the fact that the presence or absence of CVD risk factors for VTE was often not reported in detail.

In conclusion, our study provides data for patients with newly diagnosed lung cancer. Although our results did not establish a causative role of the CVD risk factors in VTE, they suggest the existence of a link between hypertension and cancer-associated VTE. Future prospective studies should further investigate the mechanism underlying this relationship.

## Patients and Methods

### Study population

From January 2009 to January 2011, unselected patients with newly diagnosed lung cancer from five hospitals that met the following inclusion criteria were included: histological confirmation of diagnosis; willingness to participate; and provided written informed consent. The exclusion criteria were as follows: any surgery, chemotherapy, or radiotherapy within the past three months; the presence of overt bacteria or a viral infection; a history of VTE (VTE diagnosis at least three months prior to recruitment); and continuous anticoagulation with vitamin K antagonists or low-molecular-weight heparins. The study was approved by the Central Ethics Committees of Beijing Chao-Yang Hospital of Capital Medical University (No. 2009-4). Data were collected from all of the patients. The methods were carried out in accordance with approved guide–lines.

### Measurement of CVD risk factors

CVD risk factors were collected at the baseline examination. The pack-years of smoking were calculated by multiplying the average number of cigarettes per day by the number of years smoked and dividing by 20. The patients were classified as never, former, or current smokers. The patients were asked to bring all of their current medications, and the medication types, including lipid-lowering medications, beta-blockers, angiotensin-converting enzyme inhibitors, and other antihypertensive medications, were recorded. Anthropometrics, including weight and height, were obtained while the patient was wearing a scrub suit. The body mass index was calculated as the weight in kilograms divided by the square of the height in meters (kg/m^2^). The blood cell counts and lipids and glucose contents were measured before initial treatment. Leukocytosis was defined as a white blood cell count (WBC) of at least 10 × 10^9^ cells/L based on the upper normal limits of a central reference laboratory. Diabetes was defined as a fasting serum glucose level of at least 7.0 mM (126 mg/dl), a non-fasting glucose level of at least 11.1 mM (200 mg/dl), or a physician diagnosis of diabetes. Hypertension was defined as a seated diastolic blood pressure of at least 90 mmHg, a systolic blood pressure of at least 140 mmHg, or a physician diagnosis of hypertension. Dyslipidemia was defined as a total cholesterol level of at least 6.22 mmol/L (240 mg/dl), a triglyceride level of at least 2.26 mmol/L (200 mg/dl), or a physician diagnosis of dyslipidemia.

### Diagnosis and classification of VTE

All of the included patients with newly diagnosed lung cancer were examined for VTE using imaging techniques within one week after admission to the hospital. Furthermore, data for all of patients for the three months prior to recruitment (medical history, presenting symptoms, diagnosis, treatment practices and follow-up data) were retrospectively collected.

DVT events were confirmed by venous ultrasound imaging or a computed tomography venous angiogram. PE events were confirmed by a computed tomography pulmonary angiogram or a ventilation-perfusion scan (if patients had renal insufficiency or allergy to contrast). All VTE events were evaluated by the independent adjudication committee^34^.

### Statistical analysis

The continuous variables were summarized as the medians with interquartile ranges or the means and standard deviations (SDs), and the differences between groups were tested with Student’s *t* test for normally distributed variables and with the Wilcoxon rank sum test for non-normally distributed variables. For the categorical variables, the percentages of patients in each category were calculated. The clinical characteristics were compared between subgroups of patients with and without VTE using the Chi-square test or Fisher’s exact test, as appropriate. Multiple logistic regression analysis was used to identify factors that were independently associated with VTE in patients with lung cancer. A *P* value of less than 0.05 was considered statistically significant. All of the analyses were performed using SPSS software for Windows (Version 19.0).

## Additional Information

**How to cite this article**: Zhang, Y. *et al*. Hypertension associated with venous thromboembolism in patients with newly diagnosed lung cancer. *Sci. Rep*. **6**, 19603; doi: 10.1038/srep19603 (2016).

## Figures and Tables

**Table 1:** Baseline demographic and clinical characteristics of the total study population (n = 632).

Characteristic	Number of patients	%
Median age, years	63.5	
25th–75th percentile	56–71	
<60	213	33.7
≥60	419	66.3
Sex		
Female	179	28.3
Male	453	71.7
Smoking		
Never	277	43.8
Former	96	15.2
Current	259	41.0
Body mass index, kg/m^2^		
Mean *(SD)*	23.3(3.4)	
Diabetes	49	7.8
Hypertension	105	16.6
Total cholesterol , mmol/L		
Mean *(SD)*	4.4(1.1)	
Triglycerides, mmol/L		
Mean *(SD)*	1.3(0.7)	
WBC count		
<10 × 10^9^/L	534	84.5
≥10 × 10^9^/L	98	15.5
Tumor histology		
Adenocarcinoma	295	46.7
Non-Adenocarcinoma	337	53.3
Squamous cell carcinoma	179	28.3
Other NSCLC	57	9.0
SCLC	101	16.0
Tumor Stage		
Localized	332	52.5
Distant metastasis	276	43.7
Unknown	24	3.8

Abbreviations: SD = standard deviation; NSCLC = non-small cell lung cancer; SCLC = small cell lung cancer; WBC = white blood cell.

**Table 2:** Comparison of demographic and clinical characteristics between patients with and without VTE.

Variable	No VTE (n = 546)	VTE (n = 86)	*p*
Age, year (%)			0.464
<60	181(33.2)	32(37.2)	
≥60	365(66.8)	54(62.8)	
Gender (%)			0.797
Female	156(28.6)	23(26.7)	
Male	390(71.4)	63(73.3)	
Smoking history (%)			0.452
Never	242(44.3)	35(40.7)	
Former	81(14.8)	15(17.4)	
Current	223(40.9)	36(41.9)	
Body mass index,kg/m^2^			0.439
Mean *(SD*)	23.2(3.5)	23.6(3.1)	
Diabetes (%)			0.285
Yes	40(7.3)	9(10.5)	
No	506(92.7)	77(89.5)	
Hypertension (%)			0.043
Yes	84(15.4)	21(24.4)	
No	462(84.6)	65(75.6)	
Total cholesterol, mol/L			0.802
Mean (SD)	4.4(1.1)	4.4(1.0)	
Triglycerides, mol/L			0.400
Mean *(SD*)	1.3(0.6)	1.3(0.9)	
WBC count (%)			0.001
<10 × 10^9^/L	472(86.4)	62(72.1)	
≥10 × 10^9^/L	74(13.6)	24(27.9)	
Tumor histology (%)			0.001
Adenocarcinoma	240(44.0)	55(64.0)	
Non-adenocarcinoma			
Squamous cell carcinoma	161(29.5)	18(20.9)	
Other NSCLC	51(9.3)	6(7.0)	
SCLC	94(17.2)	7(8.1)	
Tumor Stage (%)			0.014
Localized stage	296(54.2)	36(42.7)	
Distant Metastasis	226(41.4)	50(57.3)	
Unknown	24(4.4)	0(0)	

Abbreviations: VTE = venous thromboembolism; SD = standard deviation; NSCLC = non-small cell lung cancer; SCLC = small cell lung cancer; WBC = white blood cell.

**Table 3:** Factors associated with increased VTE risk in the multivariate logistic regression model (model 1) among newly diagnosed lung cancer patients with different tumor histologies*.

Patients Group	OR	95%CI	*P*
Hypertension			0.041
Yes	1.8	1.0–3.3	
No	1.0		
WBC count			0.001
≥10 × 10^9^/L	2.7	1.5–4.8	
<10 × 10^9^/L	1.0		
Tumor histology			0.001
Adenocarcinoma	2.3	1.4–3.8	
Non-adenocarcinoma	1.0		

^*^The variables were entered simultaneously into the multivariate logistic regression model and included age, gender, smoking, body mass index, diabetes, hypertension, dyslipidemia, WBC count, and tumor histology (adenocarcinoma vs. non-adenocarcinoma). Only variables with *P* values less than 0.05 are shown in the table.

Abbreviations: VTE = venous thromboembolism; OR = Odds Ratio; WBC = white blood cell.

**Table 4:** Factors associated with increased VTE risk in the multivariate logistic regression model (model 2) among newly diagnosed lung cancer patients with different tumor stages *.

Patients Group	OR	95%CI	*P*
Hypertension			0.029
Yes	1.9	1.1–3.4	
No	1.0		
WBC count			0.001
≥10 × 10^9^/L	2.7	1.5–4.7	
<10 × 10^9^/L	1.0		
Tumor stage			0.016
Distant metastasis	1.8	1.1–2.9	
Localized stage	1.0		

^*^The variables were entered simultaneously into the multivariate logistic regression model and included age, gender, smoking, body mass index, diabetes, hypertension, dyslipidemia, WBC count, and tumor stage (localized stage vs. distant metastasis). Only variables with *P* values less than 0.05 are shown in the table.

Abbreviations: VTE = venous thromboembolism; OR = Odds Ratio; WBC = white blood cell.

**Table 5:** Leukocytosis and ORs for VTE among newly diagnosed lung cancer patients.

Patients Group	Leukocytosis^1^	OR(95%CI)		
	Number of patients (%)	Unadjusted	Adjusted^2^	Adjusted^3^
No hypertension and no VTE	63(13.6)	1	1	1
Only hypertension	11(13.1)	0.95(0.48–1.90)	0.87(0.42–1.79)	0.90(0.43–1.88)
Only VTE	20(30.8)	2.82(1.56–5.08)	3.51(1.67–5.96)	3.21(1.69–6.08)
Both hypertension and VTE	4(19.0)	1.49(0.49–4.57)	1.33(0.41–4.39)	1.33(0.41–4.36)
*P* for trend	0.006	0.006	0.011	0.009

^1^Leukocytosis was defined as WBC count of at least 10 × 10^9^/L.

^2^Adjusted for age, gender, smoking, body mass index, diabetes, dyslipidemia, and tumor histology (adenocarcinoma vs. non-adenocarcinoma).

^3^Adjusted for age, gender, smoking, body mass index, diabetes, dyslipidemia, and tumor stage (localized stage vs. distant metastasis).

Abbreviations: VTE = venous thromboembolism; OR = Odds Ratio; WBC = white blood cell.
